# Gaming Disorders: Navigating the Fine Line between Entertainment and Addiction—Gaming History, Health Risks, Social Consequences, and Pathways to Prevention

**DOI:** 10.3390/jcm13175122

**Published:** 2024-08-29

**Authors:** George Imataka, Shu Izumi, Yuji Miyamoto, Akira Maehashi

**Affiliations:** 1 Department of Pediatrics, Dokkyo Medical University, 880 Kitakobayashi, Mibu, Shimotsuga, Tochigi 321-0293, Japan; 2 Faculty of Human Sciences, Tokyo City University, Setagaya, Tokyo 158-8557, Japan; 3 Faculty of Human Sciences, Waseda University, Tokorozawa, Saitama 359-1192, Japan

**Keywords:** behavioral addictions, gaming, gaming disorder, internet, gambling

## Abstract

The number of people immersed in excessive gaming has increased in this age of rapid digitalization. The World Health Organization and American Psychiatric Association Organization recognize a gaming disorder as a condition that results in significant problems in daily life as a result of excessive gaming. Both organizations emphasize the similarities to behavioral addictions such as gambling. We examined the appropriate usage of video games from the perspectives of health and management in this study. For the general population, video games provide positive impacts such as stress alleviation and memory improvement. Game playing leads to a loss of time and money for the individual. It also has a negative impact on the individual’s family and social life, evolving into a social problem. Gaming addiction is often accompanied by psychological disorders and other addictions, and long-term medical treatment, including approaches to the individual’s psychological background and cognitive-behavioral therapy, is necessary. Therefore, the prevention of gaming disorder is essential. From a societal standpoint, action is required in three contexts: the government, game developers, and within the household as a whole. Simultaneously, the public needs to understand the positive potential of gaming, such as e-sports.

## 1. Introduction

In Japan, invader games and other arcade games first appeared in the 1970s, followed by Nintendo’s family computer in the 1980s, and television games quickly became popular [1]. Following World War II, the core technology within digital devices evolved; electronic devices became smaller and displayed improved performance [2]. These technological advancements were applied to video game consoles. The enhanced color palette and more intricate graphics created an engaging experience for both children and adults. As a result, games become more realistic and players have penetrated the world of video games to an unprecedented level. Children may feel more compelled to play games more frequently and for longer durations. Excessive gaming, one of the components of gaming disorder, has been included in both the 11th revision of the World Health Organization (WHO)’s International Classification of Diseases (ICD-11) and the American Psychiatric Association (APA)’s Diagnostic and Statistical Manual of Mental Disorders, 5th edition (DSM-5) [3,4].

In recent years, excessive gaming has been found to negatively impact an individual’s daily life and their physical, emotional, and social welfare [5,6]. According to a meta-analysis, the worldwide prevalence of gaming disorder was 3.05% [7]. This reduced to 1.96% when only those studies that met more stringent sampling criteria were considered. Gaming disorder is more prevalent in Asian countries than in the West, at 5.08% [8], with Japan having a prevalence of 5.5%.

When arcade games became popular in the 1970s, television games were considered a barrier to home learning. Since the early days of television games, their outright prohibition or, at the very least, limited use has been advocated [8]. Supervising adults typically set time limits for children who have been exposed to video games from an early age. This is a sensible strategy since, while excessive gaming is harmful, playing video games in moderation and under supervision lowers stress [9,10]. Therefore, players may improve their lives if they approach video games with awareness. Four television game producers created the Computer Entertainment Association (CESA), which has published a note on playing video games pleasantly and safely [11]. In response to the gaming dependency being recognized by the ICD-11 and DSM-5, they advise moderate use in their note.

In this study, we aim to explore the appropriate usage of video games in the context of health and management. We emphasize the vibrant nature of the video game industry and highlight the future prospects of video games. Deterrents, related disengagement, and the economic implications of excessive gaming are elucidated and contrasted with gaming addiction. Additionally, we aim to focus on factors and recommendations for the appropriate use of video games and game addiction countermeasures adopted by organizations and game developers.

## 2. History of Games and Video Games

**Figure 1:**
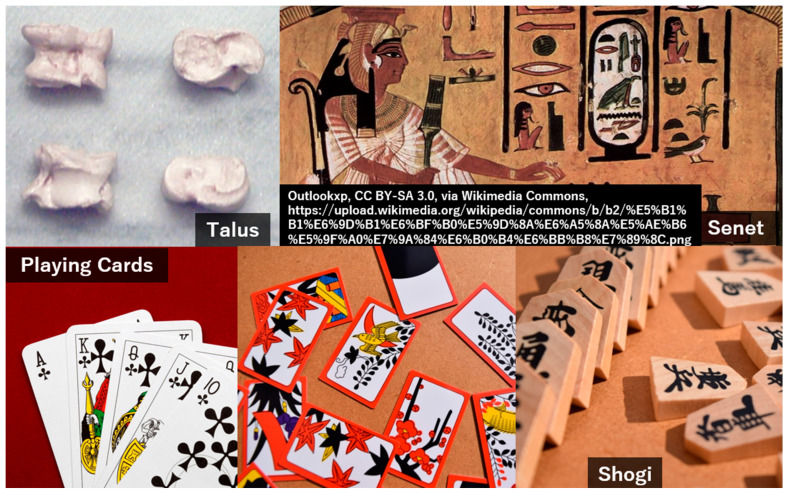
History of games: man-versus-man.

Games have been played since ancient times, as evidenced by the discovery of dice amid ancient ruins. The world’s oldest board game, Senet, the predecessor of backgammon, was also discovered in the ancient Egyptian pharaoh King Tutankhamun’s tomb. Card games then became popular among European nobility. Hana-satsu (flower tags) and shogi (Japanese chess) were then created in Japan during the Edo era. Games are historically intertwined with exposition, addiction, gambling, and betting. History is full of opulent gamblers, from Roman emperors to Marie Antoinette [12,13,14]. Table 1 summarizes the history of video games and games in general. Figure 1 represents man-versus-man games from ancient and medieval times.

**Figure 2:**
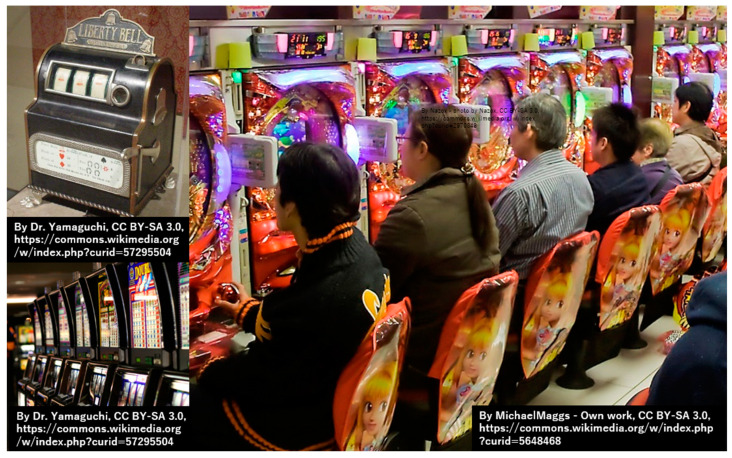
History of games: man-versus-machine.

When coin-operated slot machines were introduced around 1895, gaming developed into man-versus-machine (Figure 2). Even in present-day Japan, slot machines and pachinko are forms of man-versus-machine gambling [15,16]. Additionally, slot machines are a standard form of casino gambling in other countries. Although games originated as a form of entertainment, they have since become commercialized, with money flowing into games as players compete. The gaming industry surged to new heights with the advent of video games in the late 1950s [17]. In Japan, Space Invaders was the first arcade game that started the video game craze. Space Invaders is considered one of the most influential video games of all time. The game helped transform the video game business from a novelty to a global enterprise, ushering in the golden age of arcade video games [18].

**Figure 3:**
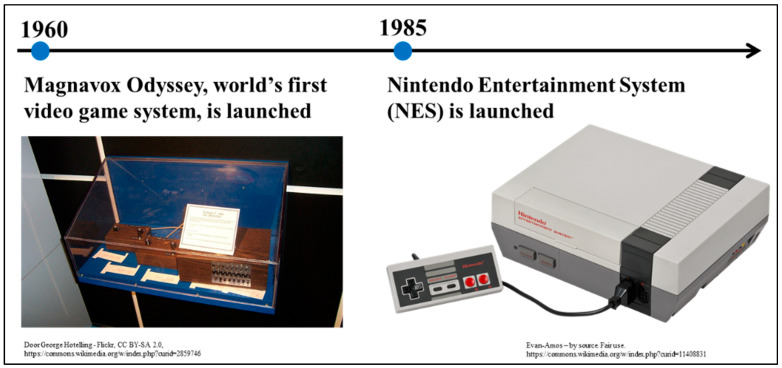
Magnavox Odyssey and Nintendo Entertainment System.

Owing to their complexity and high cost, early computers could only be used in colleges and major corporations, making them unfamiliar to the general public. Television games were developed to raise awareness of and support for computers among the general population. Ralph H. Baer focused on television games in the 1960s, alongside university game development activities. When Americans bought televisions for home use at the time, Baer saw their potential in relation to games. Following that, Baer and his colleagues created the Brown Box, the prototype for the world’s first multiplayer, multi-program video gaming system. This was released in 1972 as the Magnavox Odyssey (Figure 3). However, the gaming system was a commercial failure, selling fewer than 200,000 units [19,20]. Following that, arcade versions and home gaming devices for television games were created, although with limited success. Then, in 1985, the Nintendo Entertainment System (NES) was released (Figure 3). This marked a breakthrough in home gaming consoles. The NES employed cartridge-style software, and its main unit could be linked to a television. The Sony PlayStation and Microsoft Xbox were also later released, and these consoles also utilized the same body format, cartridge-style software, and television connectivity formats. Additionally, portable gaming devices such as the PlayStation Portable and Nintendo Game Boy were sold. However, the hardware and software were not identical to those of the NES. The 2000s saw the continuation of this format [19,21,22,23].

**Figure 4:**
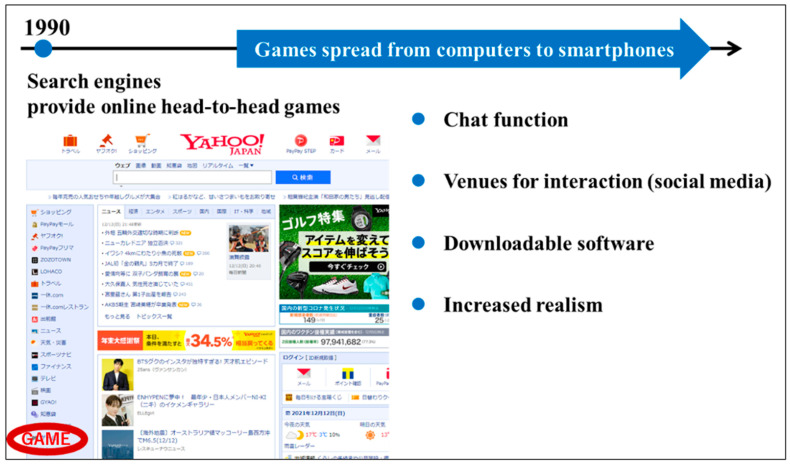
Online gaming on search engines.

During the 1990s, as the internet expanded, video games evolved yet again. Online combat games and social networking services became accessible via chat features on major search engines such as Yahoo (Figure 4). Players who have never met in person can speak while playing on the Nintendo Switch, among other modern gaming devices [23,24,25].

With the development of smartphones, mobile phone convenience eclipsed that of PCs in the 2010s [26]. Approximately 4.78 billion people (or 62.6% of the world’s population) had smartphones as of 2020. Mobile games appeared on mobile devices just as the internet did, and as smartphones gained popularity, so did games. The online gaming market was less than USD 200 billion in 2009. By 2018, the market has grown to USD 1.2 trillion, an increase of six-fold [27]. In 2020, approximately 3.1 billion people across the world played video games, which represents approximately 40% of the world’s population [28].

## 3. Gambling Addiction, Its Impact, and Countermeasures

Gambling can be defined as “betting and wagering mechanics, predominantly chance-determined outcomes, and monetization features that involve risk and payout to the player” (King DL_2015). According to the DSM-5, gambling involves risking something of value in the expectation of obtaining something of a higher value [4].

Gambling addiction is one of the behavioral and conduct-based dependencies included in the DSM-5 and is recognized as a global challenge [4]. Gambling and games such as dice and playing cards are considered to have a close relationship [29].

A US survey of gambling among adolescents and young adults reported that gambling is widespread among US youths, which is a cause of concern [30]. The cost of gambling addiction in Sweden in 2018 amounted to EUR 1.42 billion, representing 0.30% of the gross domestic product. Direct costs (medical and litigation costs) accounted for only 13% of the total costs. On the contrary, indirect costs (reduced productivity because of unemployment) accounted for more than half (59%) of total costs. Intangible costs (reduced quality of life due to mental distress) accounted for 28% of total costs. Social costs more than doubled tax revenues from gambling in 2018, resulting in more losses. The direct and indirect costs of gambling combined constitute a major social problem. The costs of gambling have reached one-third and one-sixth of the cost of smoking and drinking, respectively, in Sweden [31].

Gambling is prevalent in Japan, and addiction is a problem. Among gamblers, the most popular is the USD 200 annual per capita per lottery (calculated at JPY 100 = USD 1). The second most common form of gambling is national horse racing, participated in by 6.9% of the population aged 20 years and above. This results in an annual per capita consumption of USD 717. Other forms of public gambling appear to pose fewer challenges owing to the small number of participants. However, these estimations do not consider pachinko and pachislot, which are not classified as gambling. Advances in computer technology in the 1980s led to the use of virtual reels and random number generators to determine game results. This has led to greater payouts, more winners, and more losers. The addition of auditory effects has also contributed to the increased addictiveness. Pachinko (including pachislot) is a giant industry worth USD 230 billion in Japan, surpassing the automobile industry. Furthermore, in 2006, the amount spent on pachinko per year by Japanese people was almost identical to the annual medical expenditure [32]. This accounts for half of consumer debts.

A study published in 2021 reported that public gambling such as horse, cycling, and boat and auto racing has become more prevalent compared to pachinko games with the rise of online gambling. This allows people to participate from anywhere. Thus, an increase in online gamblers poses a great danger [33].

Several treatment options have been considered for gambling addiction. These include self-help efforts and peer support, interventions for short-term motivation, and more intensive treatment approaches. Participation in peer support programs appears optimal when combined with specialized treatment. However, participation and retention in peer support are limited. Self-directed interventions seem to only benefit some gamblers, but the involvement of face-to-face or telephonic therapist support is sought. Brief and motivational approaches can address a diverse range of problems among gamblers. Among them, cognitive behavioral therapy (CBT) has become the most common [34].

## 4. Definitions of and Factors in Gaming Disorder

### 4.1. Gaming Disorder and Symptoms Recognized by the WHO and APA

**Figure 5:**
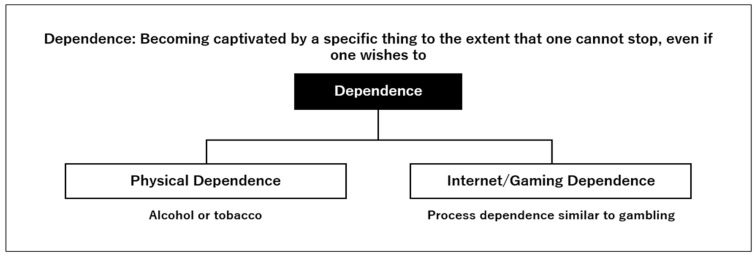
What is dependence?

The MHLW Japan defines dependency as “A state of being captivated by something specific and wanting to quit, but not being able to.” Dependency is classified into two categories (Figure 5). The first type of addiction is a state of being in a situation where things cannot be consumed or experienced in moderation and is related to substance use. The second type of addiction is a state of being too absorbed in a particular act or process and falling into a situation where nothing else can be carried out. Gaming disorder is an addiction owing to the act of immersing oneself in the game and falls under the second type of addiction. With the popularization of the internet and smartphones, internet and gaming addiction have become significant problems [4]. Therefore, countermeasures from the medical field are required.

Dependence symptoms can be broadly classified into physical dependence on substances (e.g., alcohol and tobacco) and process dependence (e.g., online games, the Internet, slot machines, pachinko, and various forms of gambling).

The DSM-5 classifies internet gaming disorder (Japanese Society of Psychiatry and Neurology Psychiatry Review Committee, 2014) as an addiction when at least one of nine clinical manifestations of five core symptoms occurs over a period of 12 months [35].

1. Being hooked on video games;
2. Experiencing unpleasant symptoms when stopping video game play;
3. Feeling a need to spend more time on video games;
4. Inability to manage video game use;
5. Losing interest in past hobbies and entertainment other than video games and feeling triggered by video games;
6. Continuing to play video games despite awareness of the related psychosocial problems;
7. Falsifying the amount of time spent on video games to family members and therapists;
8. Using video games to escape or resolve negative emotions;
9. Harming or losing relationships and work or educational opportunities because of video game use [35].

The WHO has also issued a reminder regarding the fact that internet gaming disorder is harmful behavior. To be recognized as a disorder, a pattern of behavior must be serious enough to result in significant disability in personal, family, social, educational, occupational, or other important areas of functioning and usually needs to occur continuously for at least 12 months [36].

As per ICD-11, typical symptoms of internet gaming disorder include the following [3]:

1. Impaired control over gaming (e.g., onset, frequency, intensity, duration, completion, context);
2. Gaming being prioritized over other life interests and daily activities;
3. Continuous or increasing gaming despite negative consequences.

Decreased self-esteem and social competence and feelings of loneliness are common symptoms that suggest that the condition may cause mood and anxiety disorders [37]. Conversely, mood and anxiety disorders may predispose individuals to gaming disorder [37].

Frequency of gaming, time spent on these activities, neglect of other activities and priorities, risky behavior related to gaming, negative effects of gaming, or a combination of these may increase the risk of gaming disorder and significantly increase the risk of physical or mental harm to the person or surrounding persons [3].

Additionally, physically continuing to look at the computer screen for extended periods can cause eye strain and permanent visual impairment, especially in adolescents. Prolonged and continuous sitting can lead to muscle strain and postural problems, resulting in chronic lower back and joint pain [38].

### 4.2. Neurobiological Insights and Psychological Motivations behind Gaming Disorder

From the neurobiological perspective, gaming disorder may be deeply related to habitual patterns. Studies have shown that despite the absence of exogenous chemicals, neuro-molecular and neurocircuitry components in gaming disorder are similar to substance-related dependencies [39,40,41]. Playing internet games can change brain activity and architecture in cerebral regions related to reward, motivation, memory, and cognitive control [40]. Excessive gaming is accompanied by an increase in dopamine in the dorsal striatum, which further modifies the dopamine reward pathway [39]. Then, the anterior cingulate cortex, orbitofrontal cortex, and nucleus accumbens are reconstructed [40]. Owing to these changes, natural rewards (e.g., food and sex) give reduced pleasure to an addicted gamer, further reducing behavioral control and resulting in neglect of self-care.

Results of a meta-analysis of men aged 13.8 to 25 years revealed abnormalities in activation within regions comprising the medial frontal gyrus, left cingulate gyrus, left medial temporal gyrus, and saphenous gyrus in the gaming disorder group compared to healthy controls [42]. Functional MRI studies have shown that increased activation in these areas is associated with functional impairment [43]. Hence, gaming disorder may generate reduced resistance to desire and increased impulsivity. Essentially, by examining and thinking about game clues (e.g., game images), the degree of awakening increases, rendering self-control over gaming ineffective [42].

Games being for children is an outdated notion: 68% of gamers are aged 18 years or older, and the average age of players is 30 years [44,45]. Additionally, adults tend to spend a long time on games in one session [44]. Men are likely more enthusiastic about gaming than women, and many game companies continue to develop games targeting men [40].

The motivation to play games is determined by three factors: a sense of accomplishment, sociability, and immersion [40].

Sense of accomplishment: A sense of achievement in gaming is linked to leveling up. This is derived from acquiring status and power, challenging others, and developing a feeling of competence through control. High-level and smart play helps one develop a reputation and gain admiration within the gaming community, fostering an environment wherein game players wish to spend more time and effort [40,46,47]. Studies have reported that this sense of accomplishment tends to be stronger for men than for women [48]. This may be because of the stronger desire for approval among the former.

Sociability: Many online games have features that allow players to interact. This allows them to make new friends, build new relationships, and work in teams. This can be an attractive and legitimate means for gamers, who may otherwise feel lonely, to feel accepted by society [40].

Immersion: Being absorbed in the game allows players to escape reality. In this way, players can avoid negative moods and thoughts, including fear of failure, as long as they remain in the game [40].

### 4.3. Losses Associated with Addiction and Gaming Disorder

Alexis (2019) highlights three aspects of losses incurred from substance dependency: economic cost, cost of treatment, and burden on families. We would like to discuss losses in gaming disorder from the perspective of dependency [49].

Economic Cost: The cost of purchasing drugs and alcohol, especially for those who partake on a daily basis, would be higher as the amount needed to achieve the desired effect increases. This is equivalent to betting in gambling addiction. A person with a gaming disorder would find it difficult to distinguish between gambling and gaming spending. However, excessive gaming leads to over-purchasing of charged items to advance in games.

Cost of Treatment: Bass (2015) argues that CBT is effective in treating gaming disorders. Goroll and Mulley (2014) propose using CBT as a primary or adjunct therapy for a variety of patients. This includes those with depressive disorders, generalized anxiety disorder, eating disorders, substance addiction, and chronic pain. CBT helps clients change their behavior by changing their beliefs. Withdrawal from dependency involves the application of therapies used for psychiatric disorders such as CBT. This results in a prolonged length of hospital stay and consequently high treatment costs. Alexis (2019) argues that although rehabilitation programs incur many costs, dealing with addiction becomes more difficult in the long run [39,49,50]. Additionally, they add that addicts should begin a rehabilitation program as early as possible because of the economic costs incurred until the habit is broken.

Burden on Family Members: As addiction progresses, using money to satisfy desires without paying attention to the financial burden is possible. Considering the previously mentioned economic costs, gaming places a family burden in Japan owing to billing for in-game content. According to the Consumer Affairs Agency (2014), the proportion of consultations for minors with a contract purchase amount of JPY 10 million or more related to online games increased about 3.5 times from 2009 to 2013, from 15.6% to 54.4%. The problem is that these amounts are increasing. Problems occur not only in young people but also in adults. In the case of a 25-year-old man, several years of immersion in a social game resulted in divorce because of insufficient sleep and indifference to his family members [51]. He also incurred charges of JPY 0.2 million per month.

## 5. Response of Game Developers and Gaming Groups to Gaming Disorder

### 5.1. Social Responses to Gaming Disorder

An MHLW survey showed that 4% of primary school children and 8% of middle and high school children had smartphones. Middle and high school students used smartphones for 4 h or more per day on average. One to twenty percent of junior and senior high school students already have internet gaming disorder, making gaming regulation a necessary topic of discussion in homes and schools. The Japan Pediatric Society has called for research on the opportunity costs of spending an excessive amount of time on smartphones. In Kamikawa Prefecture, a special website has been established for this purpose, and animations have been uploaded on YouTube. Moreover, this website has over 30,000 subscribers. Kagawa Prefecture implemented Japan’s first gaming regulations on 1 April 2020. The details are as follows: for gaming, 60 min but up to 90 min during the holidays; for junior high school students and younger up to 9:00 PM; for other students, up to 10:00 PM. This does not apply when smartphones are used for learning purposes. Although these regulations do not involve penalties, they have been criticized by liberalists. However, Kagawa Prefecture’s regulations have received great support in Japan as, despite the WHO classifying gaming disorder as a disease, there is no stipulated law targeting gaming addiction in the country.

### 5.2. Countermeasures against Gaming Disorder

Based on the effectiveness of tobacco warning labels, according to a systematic review, customizing in-game warnings based on the amount of time gamers spend gaming (e.g., 25+ h per week) may be a possible solution [52]. For example, the Nintendo Switch is equipped with a function to manage children’s gaming time. Moreover, game developers also need to actively promote the idea of gaming in moderation [52].

As a result, it is possible to narrow the target so that the warning is displayed to those at risk of developing a gaming disorder. Moreover, adopting self-regulation across the gaming industry has been suggested. This allows parental controls and targeted warning messages to be included in game ratings by default. In the future, information about the addictive nature of these games will be added and tested to understand how this approach can drive better game purchase decisions to protect gamers’ mental health and well-being [52].

Regarding game disorders, the preventive approach is more effective than the therapeutic approach [53,54]. Preventing the disorder (a) is inexpensive in terms of public healthcare costs, (b) reduces patient morbidity, (c) improves quality of life and well-being, (d) increases labor productivity, and (e) reduces health service utilization [55].

The Basic Act on Countermeasures for Gambling Addiction was enacted in Japan in 2018. The Act covers not only gambling but also “pachinko”. In the Act, the basic measures provided included promoting gambling addiction education and establishing specialized medical institutions, mental health counseling, and legal counseling for gambling addicts [56]. Pharmacological countermeasures included the use of antidepressants (e.g., bupropion or escitalopram), which were found to be effective in reducing the symptoms associated with online gaming [57].

In 2020, the Entertainment Software Rating Board and Pan-European Game Information introduced the “In-Game Purchases (Includes Random Items)” and the Content Descriptor “Includes Paid Random Items”, respectively. These measures were intended to help gamers make informed decisions regarding their choice of video games by flagging the random nature of certain in-game purchases. However, these measures only offer limited protection [28].

### 5.3. Response of Game Organizations to Gaming Disorder

The inclusion of gaming disorder in the ICD-11 came as a huge shock to game developers. In Japan, four organizations of game developers—the CESA, the Japan Online Game Association, the Mobile Content Forum, and the Japan e-Sports Union—have announced that they are making efforts to make games safe. The organizations recommend that users, especially minors, make rules (promises) on their own while consulting their guardians, rather than providing uniform regulations that may inhibit their diverse growth, to provide sound enjoyment of the games.

To turn safe gaming into a reality, (1) children and parents need to discuss and make rules (promises) and (2) each family should decide on limits based on its unique situation and education policy. The organizations claim to have created and disseminated materials that will provide each family with an opportunity to discuss their rules about gaming.

Additionally, games and smartphone/tablet terminals are equipped with convenient functions (parental control functions) to manage the rules set by each home. These allow detailed settings, including gaming time and internet access, availability of communication functions, the feasibility of charges, and use of game titles according to the child’s age (setting age-specific ratings). Mainly, rules should be set from childhood, and games should be used in moderation.

Conversely, the Entertainment Software Association (ESA), the largest organization of video game manufacturers in the US, opposed the WHO’s proposed definition of gaming disorder:

“Just like avid sports fans and consumers of all forms of entertainment, gamers are passionate and dedicated with their time. Having captivated gamers for more than four decades, more than 2 billion people around the world enjoy video games. The World Health Organization knows that common sense and objective research prove video games are not addictive. And putting that official label on them recklessly trivializes real mental health issues like depression and social anxiety disorder, which deserve treatment and the full attention of the medical community. We strongly encourage the WHO to reverse direction on its proposed action” [58].

Subsequently, the ESA (2019) discussed gaming disorder with the WHO and refuted the ICD-11 description. They stated that the industry would work with stakeholders, researchers, policymakers, and parents to ensure best-in-class ratings, parental controls, and other tools to help video game players and parents understand and manage healthy gaming [59].

Recent realistic games are being applied to prevent and rehabilitate cognitive function decline caused by aging and neurodegeneration. Sokolov, Collignon, and Bieler-Aeschlimann (2020) showed that placing individuals in a stimulating, highly motivating setting and providing training based on neuroscientific and neuropsychological models was more informative than conventional computer-delivered cognitive training. Raouafi andEtindele-Sosso (2017) suggested that treatment may be more effective in the face of cognitive decline and associated early symptoms. This includes memory loss and dementia of the Alzheimer’s and Parkinson’s types and medium-to-long-term memory impairments because of genetic or environmental impairments. Further, they argued for the benefits of using cognitive intelligence quotient testing and brain training through video games with practically realistic personal simulation environments [60,61].

According to the ESA (2019), about 65% of all adults in the US play video games regularly. Additionally, about 75% of households report having at least one video game player. Among American adults, the most commonly used gaming devices are smartphones (60%), PCs (52%), and gaming-only machines (49%). Similar trends have been reported in other countries. For example, in Australia, the Interactive Games and Entertainment Association (2017) reported that 67% of all Australians enjoy television gaming, and 97% of households with children engage in PC gaming. Results from studies of many American video game players have also shown positive outcomes. This includes psychomotor, cognitive, therapeutic, and educational outcomes [62,63,64]. Generally, gamers also report that gaming impacts their lives positively. Moreover, nearly 80% of all adult American gamers report that gaming leads to mental stimulation, relaxation, and stress relief. Similarly, most Australian gamers state that games lead to improved thinking (84%), dexterity (78%), and pain management (59%) [59,64,65].

Worldwide, the market for e-sports is rising, exceeding the frame of online games [66]. According to Kakehi (2017), e-sports refers to entertainment, competition, and sports played using electronic devices. It refers to watching a competition between players using computers and video games as a sporting event [66]. E-sports developed mainly in Europe, the US, and Korea in the late 1990s. The current global player population is estimated at more than 100 million, and the number of programmers whose formal occupation is e-sports is also rising.

In some countries, e-sports have already been incorporated into education. Several Swedish high schools offer three e-sports classes a week. In public high schools in Norway, e-sports is an elective subject. In China, it has been a major at state-owned universities since 2017. In Japan, in FY 2016, Tokyo Anime and Voice Dominant Specialty School opened e-Sports Professional Gamers World. In FY 2017, Hokkaido High Technology Specialty School and the Japan Institute of Engineering established an e-sports department [66].

E-sports is also attracting attention from the regional development perspective. The Japan e-Sports League is a soccer J-League game wherein local franchise teams compete in per-capita league games. Online games can be played from anywhere in Japan without any regional restrictions [66]. With the rise of e-sports under the category of gaming, an increase in problematic gaming is expected and, thus, an increase in the prevalence of gaming disorders. However, joint efforts of experts from the fields of medicine, health-related disciplines, sports science, and gaming and e-sports industries can provide support to professional players and viewers, making e-sports more fun, safer, and healthier [67,68].

**Figure 6:**
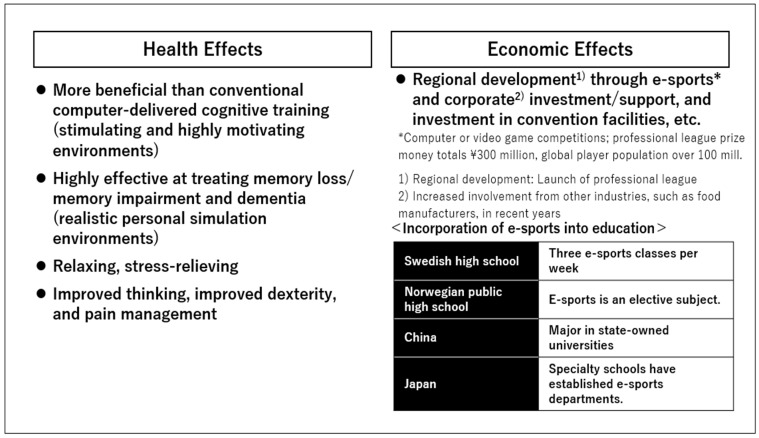
Overview of the benefits of gaming.

In recent years, evidence of the positive effects of online gaming culture on human mental health has become increasingly clear. Simultaneously, the positive effects of games on the market economy have also become apparent (Figure 6). Moreover, the theory of “gamification”, or the positive effects of games, is being explored in many countries.

## 6. Summary and Conclusions

The original purpose of gaming was competition. With the advent of computers in the 1970s, games were developed to make computers popular and encouraging engineers to learn coding. Thereafter, video games, which are now widespread in regular homes, were developed. When the internet became popular in the late 1990s, games began to use this medium. Online games enabled both traditional computer-centered matches and interaction between people from all over the world, allowing people to play at their convenience. In the 2000s, graphics improved dramatically. Mobile devices such as smartphones, then became popular. This is when excessive immersion in games was first observed and can be related to the beginnings of gaming disorder.

This period coincided with the collapse of the bubble economy, which was specific to Japan. Prior to its collapse, many salaried employees enjoyed alcohol and cigarettes together during breaks after 5:00 PM. These were called “nomination” or “nomikai” and “yannication” or “yanikai”, respectively, and were a part of the communication process among working people. However, after the bubble economy collapsed, even major companies went bankrupt. Being a hard worker was not enough to safeguard one’s job. As attachment to companies and organizations faded, competition became the norm. Opportunities for communication with peers, seniors, and juniors decreased. Additionally, tobacco was no longer generally accepted, and many smoking areas were closed. As previously discussed, the three factors that determine the motivation for playing games are the sense of accomplishment, sociability, and immersion. Immersion, particularly, gives the experience of being freed from the stresses of modern society. According to the Outline of the 2017 Patient Survey, patients with mood disorders numbered 43.3 million in 1996. By 2008, this number had more than doubled to 104.1 million. This suggests that the number of people immersed in gaming for stress relief increased rapidly (MHLW, 2017).

Addiction is a condition wherein undesirable behaviors (e.g., the consumption of alcohol and nicotine) and involvement in gambling and gaming cannot be eliminated even if individuals wish to quit them. A person who has fallen into addiction may not recognize their own destructive behavioral patterns and may need help to become free of that state. Furthermore, addiction can lead to other diseases. Treatment is expensive, and if patients belong to the working generation, the productivity of society decreases and economic losses rise exponentially. For this reason, while timely treatment for addiction is necessary, no one-size-fits-all approach exists, as with the treatment of disease. Although CBT has been proven to be effective, it is a long-term treatment. As many researchers highlight, addiction should be approached from the perspective of prevention rather than treatment. This may also be the case for gaming disorder. The fact that the WHO and APA raised the alarm about gaming disorder seems significant from the perspective of prevention.

However, the notion that video games are inherently evil is erroneous. Moderate engagement in gaming reduces stress and alleviates negative moods. Moreover, it may also be effective in preventing diseases such as dementia. In this regard, e-sports has recently been used for education and community development. Investment by prominent athletes and companies is only expected to grow. The Japanese government plans to introduce programming education in elementary schools in response to social changes such as computerization and globalization. As the internet is now part of social infrastructure, acquiring internet skills through games from early childhood has become necessary.

As many previous studies have shown, using video games appropriately is crucial. This requires adequate responses at the government, game developer, and household levels. In-game purchases have become a challenge in smartphone games in recent years, and these are highly relevant to gaming disorder. Király et al. [53] highlighted that, in addition to encouraging heavy game players, limiting game charges is necessary by setting rules such as maximum monthly or weekly charges with the game developer. Game manufacturers should, for example, warn gamers of the danger of gaming disorder on the start screen similar to warnings on cigarette packaging, and mention that they should seek help regarding the appropriate use of games. However, the benefits of the appropriate use of games should also be communicated. Awareness of the harms of video games will also be reduced by informing people that e-sports can be enjoyable. Interventions at the home level seem most effective for moderating game use. If people understand that it is not playing video games from an early age but improper use of them that is harmful, video games would be handled better.

The rapidly growing video game market is still evolving. Just as the perception of gaming disorder differs greatly between CESA and ESA, the perception of gaming differs in each country. Discussions on the use of games will need to continue worldwide.

## Figures and Tables

**Table 1:** Historical changes in the relationship between humans and games from ancient times to the present times. With the advancement of machines, the game has changed from “man versus man” to “man versus machine.

Historical Period	Relationship between Humans and Games
Ancient times	Dice made from ankle bones have been discovered in ancient ruins, and Senet—the prototype of backgammon—was discovered in the tomb of an ancient Egyptian king.
Medieval times	Betting in one-on-one games was popular using Western playing cards among the European aristocracy and pieces or cards in Asia.
1850s	The advent of the original type of slot machines brought a dynamic change from ‘man-versus-man’ to ‘man-versus-machine’.
1930s	The calculator, from which computers were derived, was developed in France.
1958	The original video game, Tennis for Two (William Higinbotham), was born.
1960s	Many engineering researchers studied computers, developing businesses, and games; the world’s first video game system, Magnavox Odyssey, was launched.
1985	Nintendo launched the Nintendo Entertainment System (NES), which became a huge hit.
1990s	Online games emerged through search engines; the realism of games increased with the ability to download them from the internet and to communicate with others through chat and social networking services.
2008 and beyond	Gaming transitioned from PCs to smartphones; in 2018, the market for online platforms was worth JPY 1.2 trillion (a six-fold increase compared with 2009).

## Data Availability

Not applicable.
